# Exploring the relationship between professional identity, cultural sensibility, and empathy among nursing students: evidence from path analysis

**DOI:** 10.1186/s12912-025-03235-1

**Published:** 2025-06-02

**Authors:** Shaimaa Mohamed Amin, Nashwa Ahmed Hussein Abdel Karim, Mahmood Ahmed Osman, Haitham Mokhtar Mohamed Abdallah, Ahmed Abdelwahab Ibrahim El-Sayed, Ahmed Farghaly Tawfik, Abeer Abd Elaziz Madian, Hanan Hosni El-Sherbini, Mohamed Hussein Ramadan Atta

**Affiliations:** 1 Community Health Nursing, Faculty of Nursing, Damanhour University, Damanhour, Egypt; 2 Psychiatric and Mental Health Nursing, Faculty of Nursing, Zagazig University, Zagazig, Egypt; 3 Zagazig University Hospitals, Zagazig University, Zagazig, Egypt; 4 Critical Care and Emergency Nursing, Faculty of Nursing, Alexandria University, Alexandria, Egypt; 5 College of Nursing, Medical Surgical Department, Jouf University, Sakaka, Saudi Arabia; 6 Nursing Administration Department, Faculty of Nursing, Alexandria University, Alexandria, Egypt; 7 Nursing Administration, Faculty of Nursing, Zarqa University, Zarqa, Jordan; 8 Nursing Administration, Faculty of Nursing, Beni-Suef University, Beni-Suef, Egypt; 9 Community Health Nursing, Faculty of Nursing, Alexandria University, Alexandria, Egypt; 10 Nursing Department, College of Applied Medical Sciences, Prince Sattam Bin Abdulaziz University, Wadi Addawasir, Saudi Arabia; 11 Psychiatric and Mental-Health Nursing Department, Faculty of Nursing, Alexandria University, Alexandria City, Egypt

**Keywords:** Empathy, Cultural sensibility, Professional identity, Nursing students, Cross-sectional study, Egypt

## Abstract

**Background:**

Empathy, cultural sensibility, and professional identity are crucial attributes for nursing students, as they significantly influence patient care quality and culturally competent practices. Understanding these traits is essential for developing tailored educational interventions to prepare nursing students for diverse healthcare environments. This study aimed to assess the levels of empathy, cultural sensibility, and professional identity among nursing students and explore the relationships among these variables.

**Methods:**

A cross-sectional descriptive design was conducted at the Faculty of Nursing, Zagazig University, Egypt. A stratified random sampling technique was used to recruit 520 undergraduate nursing students who had completed at least one clinical course. Data were collected using validated tools: the Jefferson Scale of Empathy (JSE), the Cultural Sensibility Scale for Nursing (CUSNUR), and the Professional Identity Scale for Nursing Students (PISNS). Descriptive statistics were used to characterize participants. Pearson’s correlation coefficients assessed relationships between variables. Path analysis using SPSS-AMOS version 26 evaluated the mediating role of cultural sensibility in the relationship between professional identity and empathy.

**Results:**

The mean empathy score was high (mean ± SD: 104.8 ± 15.2), with students excelling in the “Perspective Taking” subscale. The mean cultural sensibility score was moderate (mean SD: 78.6 ± 12.7), indicating room for improvement in addressing cultural diversity. Professional identity levels were strong (mean SD: 68.4 ± 10.9), with “Professional Self-Image” being the highest-rated subscale. A positive correlation was found between empathy and cultural sensibility (*r* = .42, *p* < .001) and between cultural sensibility and professional identity (*r* = .37, *p* < .001).

**Conclusion:**

This study highlights the interrelationship between empathy, cultural sensibility, and professional identity in nursing students, emphasizing the need for educational strategies that enhance these attributes to better prepare students for culturally competent and empathetic care.

**Clinical trial number:**

Not applicable.

## Introduction

In the dynamic and ever-changing landscape of healthcare, nursing students are required to cultivate a delicate balance between professional expertise, cultural awareness, and empathy to effectively meet the demands of modern patient care [1]. These elements are not only foundational to their education and practice but also serve as the cornerstone of their ability to provide compassionate, inclusive, and effective care. As future frontline caregivers, nurses must develop a professional identity that integrates technical competence with emotional intelligence, enabling them to navigate complex clinical environments while remaining patient-centered [2, 3].

Professional identity embodies the values, beliefs, and behaviors essential to nursing practice. It develops through education, clinical experiences, and mentorship, fostering qualities such as compassion and advocacy. Nursing students face challenges in integrating academic learning with clinical practice [3, 4]. A strong professional identity enhances job satisfaction and reduces burnout, emphasizing its importance in nursing education. Cultural sensibility involves recognizing and respecting diverse patient backgrounds, fostering self-reflection and emotional engagement rather than just knowledge acquisition [4, 5].

Additionally, nursing students must cultivate an awareness of their own cultural biases and develop strategies to mitigate these biases during patient interactions. This reflective practice is crucial for fostering trust and promoting inclusivity in care delivery [4]. Educational interventions aimed at enhancing cultural sensibility often include simulations, intercultural communication training, and exposure to diverse clinical settings. These experiences not only improve students’ theoretical understanding but also enable them to apply cultural awareness in real-world scenarios. Research highlights that cultural sensibility is a significant predictor of patient satisfaction and care outcomes, underscoring its importance in nursing curricula [5–7].

Empathy, essential in nursing, involves understanding and responding to patients’ emotions. It enhances communication and trust, benefiting both patients and nurses [8]. Empathy training often includes role-playing, reflective writing, and patient narratives, which help students connect with the human aspects of care. Empathy is not only beneficial for patients but also contributes to nurses’ professional satisfaction [8, 9]. It strengthens their sense of purpose and fulfillment, mitigating stress and preventing emotional detachment. However, maintaining empathy can be challenging in high-stress environments, necessitating strategies to balance emotional engagement with professional boundaries [2, 10].

The interplay between professional identity, cultural sensibility, and empathy creates a synergistic framework that influences nursing students’ readiness for practice. These elements are not isolated constructs but interdependent dimensions that collectively shape students’ attitudes, behaviors, and competencies [8, 11]. A well-developed professional identity often enhances empathy by fostering a deeper commitment to patient-centered care. Students who strongly identify with the nursing profession are more likely to internalize its values, such as compassion and altruism. This internalization enhances their capacity to empathize with patients, as they view caregiving as an integral part of their role [12, 13].

Cultural sensibility complements empathy by enabling students to understand and respect diverse patient perspectives. When nurses are culturally aware, they are better equipped to empathize with patients whose experiences and values differ from their own. This intersection is particularly vital in addressing health disparities and promoting equitable care [14, 15]. The development of professional identity is intrinsically linked to cultural sensibility. As students embrace their roles as advocates and caregivers, they recognize the importance of cultural awareness in delivering holistic care. This recognition motivates them to engage in self-reflection and continuously learn about cultural diversity [16–18]. Path analysis serves as a robust method to unravel these complex relationships, offering insights into how these constructions interact and influence each other. By mapping the pathways, this study provides a comprehensive understanding of the dynamics at play, enabling educators to design targeted interventions that address gaps and enhance synergies [9, 19].

Likewise, nursing education encounters multiple challenges in cultivating professional identity, cultural sensibility, and empathy among students [17, 20]. The growing complexity of healthcare systems, along with limited resources, can impede students’ engagement in reflective practice and meaningful interactions with patients [15, 21]. Additionally, the high-paced nature of clinical training often places greater emphasis on technical competencies at the expense of relational skills, potentially diminishing empathy. Furthermore, the interconnection between professional identity, cultural sensibility, and empathy remains a crucial research focus in nursing education. These factors not only contribute to students’ professional growth but also impact their capacity to provide compassionate, inclusive, and effective patient care [2, 22, 23]. Through path analysis, this study explores how professional identity, cultural sensibility, and empathy interconnect to shape nursing students’ practice readiness, offering insights into education strategies.

## Methods

### **Study** design and setting

This study employed a cross-sectional descriptive research design, following the Strengthening the Reporting of Observational Studies in Epidemiology (STROBE) guidelines. The research was conducted at the Faculty of Nursing, Zagazig University, located in El- Sharkia Governorate, Egypt.

### Sample size and study participants

The study included nursing students enrolled in undergraduate programs who had completed at least one clinical training course, as these students are expected to have foundational exposure to professional practice and patient interactions. Participants were required to provide informed consent and complete the study questionnaires.

To ensure sample homogeneity and control for potential confounding variables, students with prior degrees in healthcare fields were excluded. Their previous professional exposure may have shaped their professional identity and empathy differently from those entering nursing education for the first time, potentially introducing variability in the findings. Additionally, students with a documented history of psychological or emotional conditions that could significantly influence their empathy or cultural sensibility were excluded. This criterion aimed to ensure that the study primarily reflected the experiences of the general nursing student population, minimizing potential biases in the interpretation of results.

**Fig. 1:**
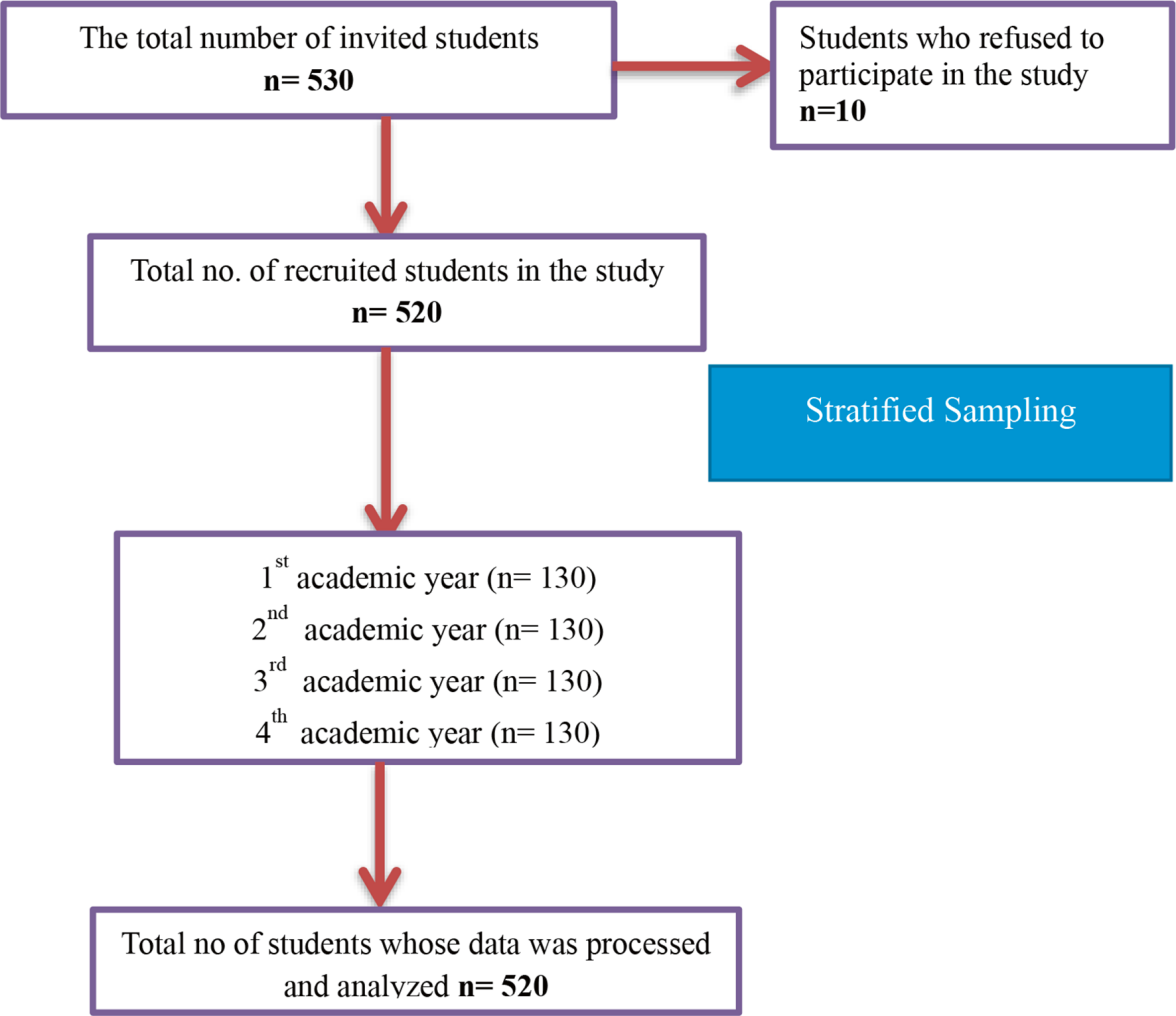
Flow chart of participants’ recruitment

The sample size for the study was determined using G*Power version 3.1.9.7 software [24]. The parameters for the calculation included an estimated effect size of 0.15, a significance level (α) of 0.05, and a desired power of 0.90 [25, 26]. Based on these specifications, the software recommended a minimum sample size of 510 participants. To account for potential participant withdrawals, dropouts, or incomplete data, the researchers opted to recruit 530 students. After accounting for ten students who declined participation, the final sample size was 520. To ensure balanced representation from all academic years, a stratified sampling technique was employed. The population was divided into strata based on academic year, and 130 students were selected from each cohort. A systematic random sampling method was then applied within each stratum, selecting every 30th student from a pre-established population list for inclusion in the study, as illustrated in Fig. 1.

### Measurements of interest

#### Demographic form

The demographic form used in this study was designed to collect important socio-demographic data from participants. It included questions on various factors such as age, sex, place of residence, family income, and employment status. These variables were selected to provide a broad understanding of the participants’ socio-economic background, which can influence various outcomes related to the research. In addition, the form gathered information on the educational levels of both the mother and father, as these factors are often correlated with educational and health behaviors. It also assessed the participants’ exposure to cultural settings and prior cultural sensibility training, recognizing the role of these experiences in shaping attitudes and behaviors.

### The ‘jefferson scale of empathy (JSE)

The Jefferson Scale of Empathy (JSE), developed by Hojat et al. (2001) [27], is specifically designed to assess empathy in healthcare professionals, including nursing students, focusing on both cognitive and emotional aspects of patient care. The scale includes 20 items across three subscales: Perspective Taking (11 items), Compassionate Care (7 items), and Walking in Their Shoes (2 items), with 10 negatively worded items that are reverse scored. Participants rate items on a 7-point Likert scale, with higher scores indicating greater empathy.

In the psychometric evaluation by Yu, Ganesh, and Lancastle (2024) [20], the JSE showed excellent validity and reliability. The Confirmatory Factor Analysis (CFA) demonstrated a good model fit, with values of χ²/df = 3.11, RMSEA = 0.062, CFI = 0.97, and TLI = 0.96, supporting the robustness of the scale’s factor structure. The Exploratory Factor Analysis (EFA) revealed a unidimensional structure with factor loadings ranging from 0.53 to 0.82, indicating that the scale effectively measures the intended construct. Furthermore, the internal consistency was high, with Cronbach’s alpha ranging from 0.80 to 0.84, confirming the scale’s reliability in assessing empathy in nursing students.

In the present study, Cronbach’s alpha was 0.88, reflecting strong internal consistency and the scale’s effectiveness in measuring empathy. To assess the construct validity, an Exploratory Factor Analysis (EFA) was conducted. Initially, factor loadings ranged from 0.442 to 0.829. However, after applying varimax rotation, the loadings improved to a range of 0.580 to 0.953. All factor loadings surpassed the 0.35 threshold, collectively explaining 68.606% of the total variance, supporting the validity of the instrument. Additionally, test-retest reliability was evaluated by administering the scale to a subset of 30 participants twice, with a two-week interval between measurements. The intraclass correlation coefficient (ICC) was 0.84, indicating strong temporal stability and reliability over time. These findings confirm the scale’s robust psychometric properties, making it a reliable and valid tool for assessing empathy in the target population.

### The cultural sensibility scale for nursing (CUSNUR)

The scale developed by Belintxon et al. (2021) [28] assesses cultural sensibility in nursing. It is a valid and reliable tool designed to measure the awareness, knowledge, and attitudes of healthcare professionals, particularly nurses, towards cultural diversity in patient care. It consisted of 22 items across four dimensions: Patient and health professional behaviours (5 items), self-assessment (4 items), Self-awareness (6 items), and cultural influence (7 items). The response to each item in the first four items was collected on a Likert scale ranging from 1 to 6 (1 = It does not influence me at all to 6 = It influences me a lot). The rest of the items were rated on a Likert scale of 1 (strongly disagree) to 6(strongly agree). A higher total score on the scale denotes a greater level of cultural sensibility in nursing. This suggests that the individual has a stronger awareness, knowledge, and understanding of cultural differences, as well as an enhanced ability to provide culturally competent care to diverse patient populations. According to Belintxon et al. (2021) [28], The Cultural Sensibility Scale (CUSNUR) demonstrated good validity and reliability. The Exploratory Factor Analysis (EFA) revealed a four-factor structure, explaining 65.69% of the variance. The factors included patient and health professional behaviors, self-assessment, self-awareness, and cultural influence. The Cronbach’s alpha for the global scale was 0.75, indicating adequate internal consistency. The model showed a good fit with a KMO value of 0.784 and Bartlett’s test result of *p* < .001, supporting the scale’s reliability and construct validity.

In the current study, Cronbach’s alpha was 0.88, indicating a high level of internal consistency and demonstrating the scale’s strong reliability in assessing cultural sensibility.

Additionally, an Exploratory Factor Analysis (EFA) was conducted to assess the scale’s validity. Before rotation, factor loadings ranged from 0.512 to 0.715, which improved to 0.552 to 0.878 after applying varimax rotation, all surpassing the 0.35 threshold. These factors collectively explained 67.547% of the total variance, supporting the scale’s validity. Moreover, the Kaiser-Meyer-Olkin (KMO) measure of sampling adequacy was 0.885, confirming that the data were appropriate for factor analysis. Furthermore, test-retest reliability was assessed using a subsample of 30 participants, who completed the scale twice with a two-week interval. The intraclass correlation coefficient (ICC) was 0.83, demonstrating strong temporal stability and further reinforcing the reliability of the instrument over time. These psychometric analyses confirm the robust reliability and validity of the translated scale, ensuring its suitability for assessing cultural sensibility in the target population.

### The professional identity scale for nursing students (PISNS)

The Professional Identity Scale for Nursing Students (PISNS), developed by Hao et al. (2014) [29], measures the professional identity of Chinese nursing students. The scale consists of 17 items across five dimensions: Professional self-image (5 items), Benefit of retention and risk of turnover (4 items), Social comparison and self-reflection (3 items), Independence of career choice (2 items), and social modeling (3 items). Each item is rated on a 5-point Likert scale, with a higher score indicating a stronger professional identity. The scale demonstrated strong reliability with a Cronbach’s alpha of 0.83, split-half reliability of 0.84, and a five-factor structure explaining 58.9% of the variance. Content validity was established through expert reviews, and construct validity was confirmed via exploratory factor analysis. Subscale Cronbach’s alpha values ranged from 0.46 to 0.83, reflecting acceptable internal consistency.

In the current study, the internal consistency reliability of the translated scales was assessed using Cronbach’s alpha, which was measured at 0.87, confirming the scale’s high reliability. To evaluate the construct validity, Exploratory Factor Analysis (EFA) was conducted. Before rotation, factor loadings ranged from 0.612 to 0.715, improving to 0.552 to 0.878 after applying varimax rotation, all exceeding the 0.35 threshold. Collectively, these factors accounted for 67.547% of the total variance, supporting the scale’s validity. Additionally, the Kaiser-Meyer-Olkin (KMO) measure of sampling adequacy was 0.885, confirming the data’s strong suitability for factor analysis. Furthermore, test-retest reliability was assessed with a subset of 30 participants, who completed the translated scales twice over a two-week interval. The intraclass correlation coefficient (ICC) was 0.82, indicating strong temporal stability and consistency over time. These findings provide robust evidence for the reliability and validity of the translated instruments, confirming their appropriateness for use in the Egyptian nursing student population.

### Study procedures

#### Tool preparation and pilot study

The research instruments, including the JSE, CUSNUR, and PISNS, were carefully translated into Arabic by bilingual experts proficient in both English and Arabic. The translations focused on ensuring cultural relevance and linguistic accuracy. The translated instruments were then back-translated into English to ensure equivalence and identify any inconsistencies. Face validity was evaluated by a panel of field experts who reviewed the instruments to ensure they accurately captured the intended constructs within the Arabic context. Additionally, feedback was gathered from potential participants to assess the clarity, relevance, and cultural appropriateness of the items, allowing for necessary refinement.

To validate the translated instruments, Exploratory factor analysis (EFA) was conducted during the pilot study. This method was selected to explore the underlying factor structure within the new linguistic and cultural setting without presuming a predefined framework. The suitability of the data for factor analysis was confirmed using the Kaiser–Meyer–Olkin (KMO) measure and Bartlett’s test of sphericity, both of which yielded satisfactory results. Reliability was evaluated through Cronbach’s alpha, demonstrating strong internal consistency and supporting the reliability of the instruments.

The pilot study involved 50 nursing students who were selected to assess the clarity, relevance, and reliability of the translated instruments (JSE, CUSNUR, and PISNS). These participants provided feedback on the tools’ cultural appropriateness and linguistic clarity, ensuring that the translated versions were understandable and contextually suitable for the Arabic-speaking population. To evaluate the internal consistency of the translated instruments, Cronbach’s alpha was (0.88,0.78 and 0.89) for JSE, CUSNUR, and PISNS respectively. The results demonstrated acceptable reliability, supporting the adequacy of the translated instruments for use in the main study. While no modifications were required based on participant feedback, this decision was informed by both qualitative assessments and statistical reliability testing, ensuring methodological rigor. Additionally, to prevent potential bias, participants from the pilot study were excluded from the main research.

### Data collection

Data collection occurred between September and November 2023, beginning with a comprehensive orientation session conducted by the researcher. During this session, the study’s objectives were outlined, emphasizing that participation was voluntary. Participants were allowed to ask questions and have their concerns addressed. Confidentiality measures were emphasized to establish trust in the research process. The questionnaires were distributed to students in diverse locations, such as lecture halls and libraries, from Sunday to Thursday between 10 a.m. and 2 p.m.

To participate, students were required to provide both verbal and written informed consent. Completing the questionnaire typically took participants 10–15 min. Throughout the process, participants were assured that their involvement was voluntary and that their responses would remain confidential. The data collection method was designed to be convenient and accessible for all participants, ensuring a smooth and efficient process. Data were collected using a structured, self-administered questionnaire distributed in hard copy format during scheduled academic sessions.

### Ethical considerations

The researcher received approval from the Research Ethics Committee of the Faculty of Nursing at Zagazig University, Egypt, under reference number (192). All procedures followed the ethical guidelines set by the Helsinki Declaration. Before participation, participants were fully informed about the study’s objectives and methods. Written informed consent was collected from all participants. The research adhered to ethical standards, ensuring voluntary participation, anonymity, and confidentiality. Participants were made aware of their right to withdraw from the study at any time without facing any consequences.

### Statistical analysis

Before data entry, all data were carefully verified for accuracy. Data analysis and tabulation were performed using IBM SPSS Statistics version 25.0. **Descriptive statistics** were employed to characterize the study participants and variables. Mean scores were calculated for numerical variables. Statistical significance was set at *p* < .05, with a significance level of *p* ≤ .001 considered highly significant. **A path analysis** model was created using SPSS-AMOS version 26 to assess mediation effects. Parametric tests were utilized as the data met the assumption of normality, confirmed through the Kolmogorov-Smirnov test, Inter-Quartile Range analysis, and examination of residual plots [30]. To address **outliers**, the Winsorizing method was applied in IBM SPSS, “Winsorizing a method used to reduce the impact of outliers by replacing extreme values outside this range (Q1–1.5 IQR to Q3 + 1.5 IQR) with the nearest value within the acceptable range” [43]. **Multicollinearity “**that is, A high correlation/relationship between independent variables with one potentially being linearly predicted from the others” [41] among variables was assessed. Tolerance values exceeded 0.1, and the Variance Inflation Factor (VIF) for all variables, including professional identity and cultural sensibility, remained below the threshold of 3 (VIF < 1.0), indicating no significant multicollinearity. **Homoscedasticity** “refers to Equal spread of one variable around all the levels of another variable” [42] was verified through an examination of standardized residual plots. The fulfilment of all assumptions ensures the robustness of our findings. Pearson’s correlation coefficients were calculated to analyze bivariate relationships between the study variables and their sub-dimensions.

## Results

**Table 1:** Distribution of the study participants according to their characteristics (*n* = 520)

Variable	Category	Frequency	Percent
Age	< 20	78	15.0
	20-	328	63.1
	23+	114	21.9
Mean ± SD		21.7 ± 2.7	
Gender	Male	88	16.9
	Female	432	83.1
Residence	Rural	385	74.0
	Urban	135	26.0
Occupation	Not-working	359	69.0
	Part-time	130	25.0
	Full-time	31	6.0
Family income	Enough	88	16.9
	Enough & Save	84	16.2
	Not-Enough	348	66.9
Mother’s educational level	Illiterate	55	10.6
	Basic education	84	16.2
	Secondary education	184	35.4
Father’s Educational Level	University & above	197	37.9
	Illiterate	38	7.3
	Basic education	85	16.3
	Secondary education	165	31.7
	University & above	232	44.6
Prior cultural sensitivity training	Yes	315	60.6
	No	205	39.4
Experience with diverse cultures	Never	30	5.8
	Rarely	62	11.9
	Sometimes	244	46.9
	Often	131	25.2
	Very often	53	10.2
	Total	520	100.0

Table 1 provides a descriptive overview of the sociodemographic characteristics of the study participants (*n* = 520). The sample is predominantly female (83.1%) and resides primarily in rural areas (74.0%). Most participants are young adults, with 63.1% aged 20–23 and a mean age of 21.7 years. A significant proportion (69.0%) are not employed and experience financial insufficiency (66.9%). Regarding educational background, a notable proportion of participants’ mothers have at least a secondary education (35.4%), while a higher proportion of fathers have a university education (44.6%). Sixty-point-6% (60.6%) of participants have undergone prior cultural sensitivity training. Concerning exposure to diverse cultures, 46.9% of participants reported “sometimes” and 25.2% “often” encountering diverse cultural environments.

**Table 2:** Descriptive statistics and pairwise correlations of the study variables and their dimensions (*n* = 520)

		1	2	3	4	5	6	7	8	9	10	11	12	13	14	15
1	**Empathy**															
2	Perspective Taking	0.391**														
3	Compassionate Care	0.522**	-0.085													
4	Walking Shoes	0.311**	0.155**	0.225**												
5	**Cultural Sensibility**	-0.032	0.038	− 0.141**	0.031											
6	Patient health Professional behaviors	-0.036	0.042	− 0.136**	0.037	0.553**										
7	Self-assessment	− 0.174**	0.062	− 0.355**	-0.045	0.514**	0.164**									
8	Self-awareness	0.041	0.013	0.063	0.061	0.707**	0.201**	0.051								
9	Cultural influence	0.039	-0.007	-0.030	0.007	0.705**	0.179**	0.255**	0.287**							
10	**Professional Identity**	0.017	0.292**	− 0.184**	0.040	-0.007	-0.012	0.270**	− 0.162**	-0.025						
11	Professional self-image	0.052	0.174**	− 0.131**	0.054	-0.029	-0.048	0.237**	− 0.143**	-0.047	0.748**					
12	Retention turnover	0.052	0.277**	− 0.139**	0.071	-0.008	-0.039	0.186**	− 0.094*	-0.022	0.813**	0.581**				
13	Self-reflection	-0.023	0.290**	− 0.201**	-0.006	0.002	-0.001	0.174**	− 0.095*	-0.018	0.748**	0.491**	0.577**			
14	Career choice	0.007	0.167**	-0.058	0.097*	0.034	0.013	0.194**	-0.049	-0.022	0.487**	0.402**	0.324**	0.379**		
15	Social modeling	0.076	0.254**	− 0.142**	0.069	-0.011	-0.021	0.231**	− 0.093*	-0.080	0.669**	0.434**	0.575**	0.526**	0.293**	
**Mean + SD**		90.3 ± 7.8	62.4 ± 5.7	21.6 ± 8.9	8.5 ± 2.6	68.6 ± 13.9	13.8 ± 4.5	16.4 ± 4.6	16.6 ± 7.0	21.8 ± 5.8	67.8 ± 10.0	18.9 ± 4.3	15.8 ± 2.5	12.4 ± 1.9	7.7 ± 1.8	13.2 ± 1.8

Table 2 presents descriptive statistics and pairwise correlations among the study variables and their dimensions. The table shows that Empathy has moderate to strong positive correlations with perspective-taking (*r* = .391) and Compassionate Care (*r* = .522). “Walking Shoes” demonstrates moderate positive correlations with Perspective Taking (*r* = .155), Compassionate Care (*r* = .225), and Empathy (*r* = .311). Several significant correlations are observed among the dimensions of professional identity. Professional Identity is positively correlated with Professional Self-Image (*r* = .748), Retention Turnover (*r* = .813), Self-Reflection (*r* = .748), Career Choice (*r* = .487), and Social Modeling (*r* = .669). Similarly, Retention Turnover shows strong correlations with Professional Self-Image (*r* = .813), Self-Reflection (*r* = .748), and Social Modeling (*r* = .669).

**Table 3:** Correlations between study variables and study participants’ characteristics (*n* = 520)

Participants’ characteristics	Empathy	Cultural sensibility	Professional identity
Age	− 0.107*	-0.030	0.085
Gender	0.047	− 0.111*	-0.028
Place of residence	0.029	0.032	0.001
Occupation	0.051	0.122**	− 0.134**
Family income	0.018	-0.019	-0.082
Mother’s educational level	0.021	-0.060	-0.078
Father’s Educational Level	-0.069	0.061	0.000
Prior cultural sensitivity training	0.152**	-0.043	− 0.087*
Experience of diverse cultures	− 0.104*	0.057	0.022

**. Correlation is significant at the 0.01 level (2-tailed)

*. Correlation is significant at the 0.05 level (2-tailed)

Table 3 explores the correlations between study variables (Empathy, Cultural Sensibility, and professional identity) and participants’ characteristics. Several significant relationships are observed. Age is negatively correlated with Empathy (*r* = −.107), suggesting that younger participants may exhibit higher levels of empathy. Interestingly, Gender is negatively correlated with Cultural Sensibility (*r* = −.111), indicating that males in this sample may demonstrate lower levels of cultural sensibility compared to females. Occupation shows a significant positive correlation with Cultural Sensibility (*r* = .122) and a negative correlation with Professional Identity (*r* = −.134). Prior cultural sensitivity training is positively associated with Empathy (*r* = .152), suggesting that such training may contribute to increased empathy levels. Conversely, greater exposure to diverse cultures is negatively correlated with Empathy (*r* = −.104). These findings highlight the complex interplay between individual characteristics and the development of empathy and cultural sensitivity.

**Table 4:** Path analysis of direct and indirect effects of professional identity on empathy mediated by cultural sensibility

Variable 1	Direction	Variable 2	β	S.E.	C.R.	*P*	Significance
Cultural Sensibility	<---	Professional Identity	− 0.010	0.545	− 0.202	0.840	insignificant
Empathy	<---	Cultural Sensibility	0.282	0.014	2.685	0.007	
Empathy	<---	Professional Identity	0.987	0.223	6.887	***	significant
Social modeling	<---	Professional Identity	0.692				significant
Career choice	<---	Professional Identity	0.467	0.073	9.509	***	significant
Self-reflection	<---	Professional Identity	0.738	0.081	14.353	***	significant
Retention turnover	<---	Professional Identity	0.803	0.110	15.193	***	significant
Professional self-image	<---	Professional Identity	0.689	0.179	13.561	***	significant
Perspective Taking	<---	Empathy_	0.333				significant
Compassionate Care	<---	Empathy_	− 0.231	0.256	-4.230	***	significant
Walking with their shoes	<---	Empathy_	0.076	0.066	1.599	0.110	insignificant

**β**: Standardized Coefficients, **S.E.**: standard error, **C.R**.: Critical ratios, *: Statistically significant at *p* ≤ .05

**Fig. 2:**
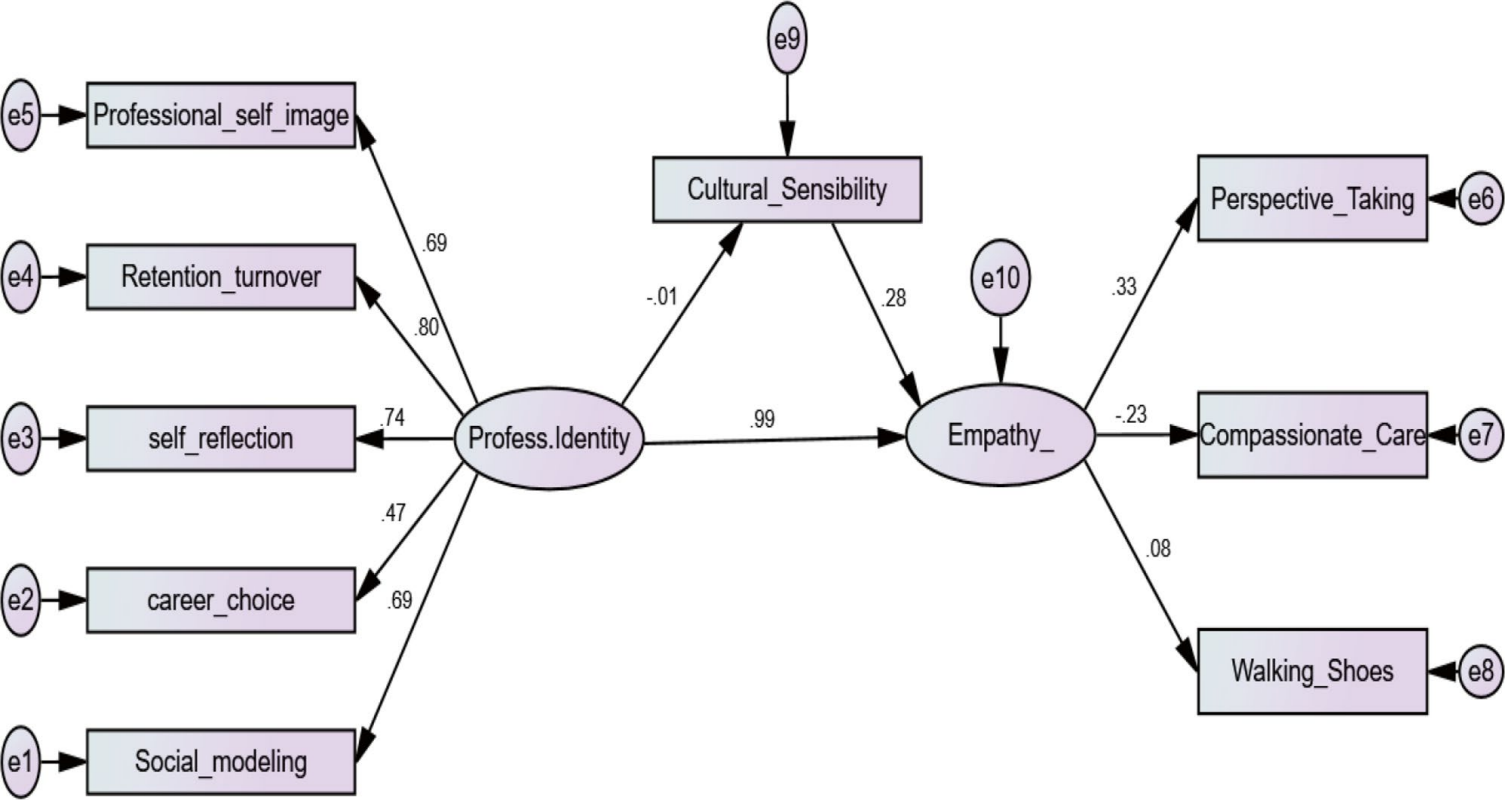
Path analysis of direct and indirect effects of professional identity on empathy mediated by cultural sensibility. Model fit parameters X^2^/DF, GFI, CFI; RMSEA (3.84, 0.962, 0,925, 0.074). GFI = Goodness of Fit Index, CFI = Comparative fit index, and RMSEA = Root Mean Square Error of Approximation. Model Chi square = 95.96, DF = 25, *p* < .000*

Table 4; Fig. 2 present the results of the path analysis, illustrating standardized regression weights, standard errors (SE), critical ratios (CR), and significance (p-values) for both the direct and indirect effects of professional identity on empathy. The statistical analysis was conducted using SPSS-AMOS. The model fit indices indicate a good model fit, with the comparative fit index (CFI) at 0.925, the root mean square approximation error (RMSEA) at 0.074, and the chi-square/degrees of freedom ratio (χ²/df) at 3.84. CFI of (> 0.90 indicates a good fit), and RMSEA (< 0.08 indicates an acceptable fit) [25], “this reflection of model fit parameters as acceptable based on literature as mentioned, but for integrity other literature view this parameters specially CFI and RMSEA as mediocre” The results of the path analysis demonstrate a significant positive impact of both professional identity and cultural sensibility on empathy (*p* < .001 for both). However, cultural sensibility does not mediate the relationship between professional identity and empathy, as the direct effect of professional identity on cultural sensibility is not statistically significant.

**Table 5:** Regression analysis of relationships between cultural sensibility, professional identity & socio-demographics of study participants, and their empathy (*n* = 520)

	Unstandardized coefficients		Standardized coefficients	t	Sig.	Collinearity statistics	
	B	Std. Error	Beta			Tolerance	VIF
(Constant)	91.084	4.131		22.050	0.000		
Cultural Sensibility	-0.069	0.025	-0.115	-2.779	0.006	0.997	1.003
Professional Identity	-0.229	0.035	-0.275	-6.614	0.000	0.986	1.014
Prior cultural sensitivity training	2.036	0.708	0.119	2.875	0.004	0.989	1.011

a. Dependent Variable: Empathy

Model ANOVA, F = 17.65, Sig.= 0.000, *R* = .347, R Square = 0.121

Table 5 presents the results of a multiple linear regression analysis examining the relationships between empathy (the dependent variable) and cultural sensibility, professional identity, and prior cultural sensitivity training (the independent variables). The model, which is statistically significant (F = 17.65, *p* < .000), explains 12.1% of the variance in empathy scores (R²=0.121). Both cultural sensibility (B=-0.069, *p* = .006) and professional identity (B=-0.229, *p* < .000) show statistically significant *negative* relationships with empathy, suggesting that higher levels of these variables are associated with *lower* empathy scores. Conversely, prior cultural sensitivity training (B = 2.036, *p* = .004) demonstrates a statistically significant *positive* relationship with empathy, indicating that individuals with prior training report higher empathy. The collinearity statistics (Tolerance and VIF) suggest that multicollinearity is not a significant issue in this model. The constant (intercept) of the model is 91.084.

## Discussion

This study examined the relationships between professional identity, cultural sensibility, and empathy among nursing students, providing valuable insights into how these variables contribute to the development of empathetic competencies. The findings revealed significant positive associations between professional identity, cultural sensibility, and empathy, emphasizing their individual and collective importance in nursing education and practice. Additionally, demographic factors, including gender, age, prior cultural training, and exposure to diverse cultures, significantly influenced the levels of empathy and cultural sensibility, further highlighting the complex interplay of these factors in shaping nursing students’ capabilities.

### Professional identity and empathy

The path analysis demonstrated that professional identity was a strong predictor of empathy among nursing students. This suggests that students with a well-established professional identity may exhibit higher levels of empathy due to their deeper commitment to patient-centered care, ethical responsibilities, and professional values. Nursing students with a stronger professional self-image may be more inclined to adopt patient advocacy roles and demonstrate higher empathy levels in clinical interactions [12]. From a practical perspective, this finding suggests that nursing education should place greater emphasis on strategies that strengthen professional identity, such as mentorship programs, reflective practice, and role modeling by experienced nurses.

This finding aligns with research by Sang et al. (2022) [31], who identified professional identity as a critical factor influencing nursing students’ readiness for empathetic interactions. Baseer et al. (2024) similarly emphasized professional identity as a predictor of empathy in healthcare professionals, suggesting that identity development equips students with the confidence and self-awareness needed to prioritize patient-centered care. Moreover, Wang et al. (2024) [32] underscored the importance of reflective practices in strengthening professional identity and fostering empathy. However, divergent findings, such as those reported by Dahm & Yates (2020) [13], who observed no significant relationship between professional identity and empathy, indicate that cultural and contextual factors may influence this association and warrant further investigation.

### Cultural sensibility and empathy

Cultural sensibility also emerged as a significant determinant of empathy, highlighting its role in enhancing nursing students’ ability to understand and respond to the diverse needs of patients. Students with higher cultural sensibility demonstrated greater empathy, particularly in their capacity for perspective-taking and compassionate care. This finding supports the work of Belintxon et al. (2021) [28], who identified cultural sensibility as a foundational competency for providing empathetic and culturally appropriate care. Similarly, Erden et al. (2023) [7] and Zarei et al. (2019) [9] emphasized the importance of cultural competence in fostering empathy, particularly in multicultural healthcare settings.

These results underscore the necessity of integrating cultural training and diversity-focused education into nursing programs. Possible approaches include case-based learning, cultural immersion experiences, and interprofessional workshops that encourage critical reflection on cultural biases and promote inclusive practices [33, 34]. However, Butte and Hristova (2024) [23] reported limited effectiveness of cultural training on empathy, which they attributed to inconsistent program designs and a lack of practical application in clinical contexts. This suggests that cultural training must be carefully structured and evaluated to maximize its impact on empathetic competencies.

### Professional identity and cultural sensibility

The relationship between professional identity and cultural sensibility further highlights the interconnectedness of these variables in shaping nursing students’ approaches to patient care. Our study found a positive correlation between professional identity and cultural sensibility, suggesting that as nursing students develop a stronger sense of professional identity, they also become more culturally sensitive. This relationship underscores the importance of integrating cultural competence into professional identity formation to ensure that future nurses are both ethically grounded and culturally competent in their practice.

Professional identity provides a foundation for understanding ethical responsibilities, guiding students in developing a strong commitment to patient-centered care [15]. At the same time, cultural sensibility equips students with the necessary tools to navigate cultural differences effectively, promoting inclusivity and reducing biases in clinical interactions [20]. The positive correlation observed in our study suggests that students who internalize nursing values and see themselves as part of the profession are more likely to recognize the importance of cultural awareness and apply it in their care. This finding aligns with research by Sagarra-Romero et al. (2024) [15] and Gradellini et al. (2021) [35], which highlights the benefits of combined training approaches in fostering both professional and cultural competencies.

The practical implications of this relationship are significant for nursing education. A well-developed professional identity may enhance students’ motivation to engage in culturally competent care, as they view inclusivity and respect for diversity as integral to their professional role. Conversely, exposure to diverse cultures and structured cultural competence training may reinforce students’ professional identity by broadening their understanding of nursing as a global and patient-centered discipline. This bidirectional influence suggests that nursing curricula should integrate professional identity development with cultural training rather than treating them as separate components.

Wang et al. (2022) [32] emphasized the importance of comprehensive curricula that link these constructs, arguing that such integration is essential for preparing nurses to meet the needs of increasingly diverse patient populations. To achieve this, nursing programs should incorporate case-based learning, role-playing scenarios, and immersive cultural experiences that allow students to apply professional values in diverse healthcare settings. Additionally, mentorship programs and reflective exercises can help students understand how cultural competence enhances their professional growth and ethical practice.

### The interplay between study variables

The results revealed significant direct effects of professional identity and cultural sensibility on empathy, reinforcing their roles as independent predictors of empathetic behaviors among nursing students. Professional identity demonstrated the strongest direct effect on empathy, highlighting its foundational role in fostering emotional connections with patients through enhanced self-image, reflective practices, and role clarity. Similarly, cultural sensibility directly influences empathy by fostering greater awareness and adaptability to cultural diversity, which are critical for patient-centered care.

A key finding of this study was that cultural sensibility did not mediate the relationship between professional identity and empathy. This suggests that cultural sensibility and professional identity may operate as independent pathways influencing empathy rather than interacting hierarchically. Several possible explanations can be considered. First, cultural sensibility and professional identity may develop through separate educational and experiential processes. While professional identity is often cultivated through role modeling and internalization of nursing values, cultural sensibility may require targeted exposure to diverse patient populations and structured cultural competence training [10]. The lack of mediation suggests that the mechanisms linking these variables to empathy are not necessarily interdependent.

Second, if cultural training is not fully embedded within the broader process of professional identity formation, students may not perceive cultural sensibility as an intrinsic component of their nursing role. Some studies suggest that cultural competence training is most effective when combined with experiential learning and reflective practice rather than being presented as a standalone component [22, 23]. This could explain why cultural sensibility did not significantly influence the professional identity-empathy link in this study. Third, the influence of cultural sensibility on empathy may vary depending on the diversity of clinical exposure students receive. In homogeneous cultural environments, students may not have sufficient opportunities to apply cultural sensibility skills in meaningful ways, reducing their overall impact on empathy development [11]. Future research should explore whether increased exposure to multicultural patient populations enhances the role of cultural sensibility in fostering empathy.

Finally, the lack of mediation may indicate that other variables, such as emotional intelligence or resilience, play a more significant role in bridging the relationship between professional identity and empathy. Studies have suggested that these factors contribute to nurses’ ability to manage emotional demands while maintaining a strong professional commitment [7, 31]. Future research should investigate whether these constructs act as alternative mediators in the professional identity-empathy relationship.

This suggests that professional identity and cultural sensibility contribute to empathy through parallel or context-specific pathways rather than interdependent mechanisms. Such results align with the findings of Ouedraogo (2021) [14], who emphasized the complementary but independent effects of these constructs on empathy development. While previous studies, such as Soleimani & Yarahmadi (2023) [16], have suggested potential synergistic relationships between cultural and professional competencies, the absence of mediation in this study highlights the need for further research into how these constructs dynamically interact in various educational and clinical contexts.

The findings highlight the need for nursing education programs to adopt a dual approach: strengthening professional identity while enhancing cultural sensibility through immersive experiences. Strategies such as interdisciplinary cultural competency workshops, patient-centered simulations, and structured reflection exercises may help integrate these constructs more effectively. Furthermore, longitudinal studies examining the long-term impact of professional identity and cultural sensibility on empathy development could provide deeper insights into their dynamic interactions.

### Role of demographic characteristics in the interplay between study variables

Demographic variables provided additional insights into how the interconnection between professional identity, cultural sensibility, and empathy may be influenced by individual characteristics. Female students demonstrated higher levels of cultural sensibility and empathy, which may stem from societal norms that emphasize emotional labor and caregiving roles among women. Younger students exhibited higher empathy levels, possibly due to their recent exposure to patient-centered educational practices and greater openness to reflective learning. Furthermore, prior cultural training significantly enhanced both cultural sensibility and empathy, suggesting that experiential learning fosters a deeper appreciation of diversity and emotional intelligence.

These demographic influences may moderate the relationships between professional identity, cultural sensibility, and empathy, shaping how these constructions develop and interact in nursing students. Supporting evidence from studies like Valdez et al. (2021) [19] and El-Sayed et al. (2024) [36] highlights the role of gender and experiential factors in enhancing empathy and cultural competence. However, contradictory findings by Liao & Wang (2020) [37] suggest that demographic effects may vary across populations, emphasizing the importance of tailoring educational strategies to specific student groups to optimize the interplay of these critical variables.

Gender differences in cultural sensibility among nursing students, particularly the lower scores observed among male students, warrant deeper examination within the socio-cultural context of Egypt. In patriarchal societies, traditional gender roles often position women as primary caregivers, fostering stronger emotional engagement and cultural awareness, which may explain their higher cultural sensibility scores [38]. Conversely, male students may have fewer opportunities or societal expectations to engage in culturally reflective practices, leading to a gap in cultural competence [39].

This aligns with previous research indicating that gendered socialization influences attitudes toward empathy and diversity in healthcare [40]. Addressing this disparity in nursing education requires targeted interventions, such as structured cultural competence training tailored to male students, mentorship programs led by culturally competent male nurses, and increased exposure to diverse patient populations through clinical rotations. By integrating these strategies, nursing curricula can ensure that all students, regardless of gender, develop the cultural awareness necessary for patient-centered and inclusive care.

Overall, the findings of this study underscore the importance of professional identity and cultural sensibility as key drivers of empathy in nursing students. These variables should be prioritized in educational strategies to prepare students for the emotional and cultural complexities of patient care. The significant influence of demographic factors further highlights the need for tailored interventions that account for individual differences in learning and development. Future research should explore innovative approaches to integrating professional and cultural training while examining the contextual factors that shape their relationships with empathy.

### Unexpected relationship between cultural sensibility and empathy

An unexpected finding in this study was the negative association between cultural sensibility and empathy (B = -0.069, *p* = .006). This result contrasts with the theoretical framework and prior research, which generally suggest that cultural sensibility enhances empathy by fostering a deeper understanding of diverse patient backgrounds. However, this inverse relationship may be explained by context-specific factors.

One possible interpretation is that as nursing students develop higher cultural sensibility, they may become more aware of cultural differences and the complexities of intercultural interactions. This heightened awareness could lead to increased self-consciousness, hesitation, or fear of unintentionally offending patients from different cultural backgrounds, potentially inhibiting the spontaneous expression of empathy. Prior studies have highlighted that early stages of cultural competence development can sometimes lead to uncertainty or discomfort in cross-cultural communication, which might temporarily impact empathetic responses.

Another explanation could be related to academic and clinical exposure. Nursing students in this study may have limited direct patient interactions with culturally diverse populations, meaning their cultural sensibility is primarily shaped by theoretical knowledge rather than real-world experiences. Without practical reinforcement, increased cultural awareness might not translate directly into higher empathetic engagement but rather contribute to cognitive overload or emotional distancing.

To further explore this finding, future research should employ longitudinal designs to examine whether this negative relationship shifts over time as students gain more clinical exposure and confidence in cross-cultural interactions. Additionally, qualitative studies could provide deeper insights into students’ perceptions of cultural sensibility and how it influences their empathetic behaviors in patient care settings.

Despite this unexpected result, this study contributes to the growing body of knowledge on the complex interplay between cultural sensibility and empathy, emphasizing the need for nursing curricula that integrate experiential learning, intercultural communication training, and structured reflection to help students translate cultural awareness into enhanced empathetic practice.

### Implications

Nursing education should prioritize integrating cultural sensibility and empathy training into the curriculum to prepare students for diverse healthcare environments. Structured programs incorporating simulations, case studies, and role-playing can help nursing students develop essential patient-centered care skills in multicultural settings. Additionally, fostering a strong professional identity through mentorship, reflective practices, and engagement in professional organizations is crucial for career development and ethical practice.

Gender-sensitive educational strategies must be embedded within nursing programs to address the distinct learning needs of male and female students, ensuring equitable opportunities for professional growth. Interdisciplinary collaborations, including partnerships with sociology and psychology experts, can further enrich students’ understanding of cultural nuances and enhance their ability to navigate complex social dynamics in healthcare.

From a research perspective, future studies should examine mediating factors such as emotional intelligence, resilience, and communication skills in the relationship between professional identity, cultural sensibility, and empathy. Longitudinal research is essential to assess the long-term impact of educational interventions and their effectiveness in shaping professional identity and empathetic practice.

In clinical practice, healthcare institutions should implement continuous professional development programs focused on cultural competence and empathy-building. Hospitals and healthcare facilities can benefit from interdisciplinary training workshops and mentorship programs that reinforce ethical decision-making and patient advocacy. Additionally, workplace policies should support diverse teams through targeted initiatives, such as inclusive leadership training and culturally adaptive care models, ultimately enhancing nurses’ ability to provide equitable, patient-centered care and improve health outcomes.

### Strengths and limitations

This study employed a systematic random sampling method to ensure a balanced representation across academic years, providing a comprehensive view of the nursing student population. The use of validated tools, including the JSE, CUSNUR, and PISNS, with strong psychometric properties further strengthened the reliability of the findings. Additionally, the pilot study ensured the cultural and linguistic relevance of the instruments, reflecting meticulous preparation. However, the study’s cross-sectional design limits its ability to establish causal relationships, as it captures data at a single point in time rather than tracking changes over time. Future longitudinal studies are recommended to better understand the directionality of relationships among the variables. Additionally, the sample is disproportionately female, predominantly from rural areas, and includes a high percentage of financially strained students. This lack of diversity limits the generalizability of the findings to the broader nursing population in Egypt and beyond. This limits the generalizability of the findings to the broader nursing population in Egypt and beyond. Future research should consider more diverse and geographically varied samples to enhance external validity.

Furthermore, the study was conducted at a single institution, which may restrict the generalizability of findings to nursing programs in different educational and cultural contexts. Future research should consider multi-institutional or cross-cultural studies to enhance external validity. While the exclusion criteria ensured a more homogeneous sample, they may have limited the applicability of the results to a broader nursing student population, particularly in terms of gender diversity and prior healthcare experience. Despite these limitations, this study provides valuable insights into the interplay of empathy, cultural sensibility, and professional identity among nursing students, offering a foundation for future research and practical implications in nursing education.

## Conclusion

The study highlights the significant influence of professional identity and cultural sensibility on empathy among nursing students, emphasizing the direct relationship between professional identity and empathy. While cultural sensibility positively impacts empathy, it does not mediate the relationship between professional identity and empathy. The findings also underscore the role of sociodemographic factors, such as prior cultural sensitivity training, gender, and occupational status, in shaping empathy and cultural sensibility levels.

These results call for targeted interventions, including cultural competence training and strategies to strengthen professional identity, to enhance empathy in nursing education and practice. Incorporating structured programs into nursing curricula that promote reflective practice, intercultural experiences, and mentorship could foster professional identity and empathy development. Additionally, healthcare institutions can utilize these findings to inform continuing education initiatives aimed at cultivating compassionate, culturally sensitive care. Ultimately, this contributes to improved patient satisfaction, therapeutic relationships, and more equitable healthcare delivery.

## Data Availability

The datasets generated and analyzed during the current study are not publicly available due to confidentiality agreements but are available upon reasonable request from the corresponding author.
